# High Expression of STAT3 in Subcutaneous Adipose Tissue Associates with Cardiovascular Risk in Women with Rheumatoid Arthritis

**DOI:** 10.3390/ijms18112410

**Published:** 2017-11-13

**Authors:** Mitra Nadali, Rille Pullerits, Karin M. E. Andersson, Sofia Töyrä Silfverswärd, Malin C. Erlandsson, Maria I. Bokarewa

**Affiliations:** 1 Department of Rheumatology and Inflammation Research, Institution of Medicine, Sahlgrenska Academy at University of Gothenburg, 413 46 Gothenburg, Sweden; mitra.nadali@rheuma.gu.se (M.N.); rille.pullerits@rheuma.gu.se (R.P.); karin.andersson@rheuma.gu.se (K.M.E.A.); sofia.silfversward@rheuma.gu.se (S.T.S.); maria.bokarewa@rheuma.gu.se (M.I.B.); 2 Rheumatology Clinic, Sahlgrenska University Hospital, 413 45 Gothenburg, Sweden; 3 Department of Clinical Immunology and Transfusion Medicine, Sahlgrenska University Hospital, 413 46 Gothenburg, Sweden

**Keywords:** rheumatoid arthritis, cardiovascular risk, adipose tissue, STAT3, IL6, leptin

## Abstract

Despite the predominance of female patients and uncommon obesity, rheumatoid arthritis (RA) is tightly connected to increased cardiovascular morbidity. The aim of this study was to investigate transcriptional activity in the subcutaneous white adipose tissue (WAT) with respect to this disproportionate cardiovascular risk (CVR) in RA. CVR was estimated in 182 female patients, using the modified Systematic Coronary Risk Evaluation scale, and identified 93 patients with increased CVR. The overall transcriptional activity in WAT was significantly higher in patients with CVR and was presented by higher serum levels of WAT products leptin, resistin and IL-6 (all, *p* < 0.001). CVR was associated with high WAT-specific transcription of the signal transducer and activator of transcription 3 (*STAT3*) and the nuclear factor NF-kappa-B p65 subunit (*RELA*), and with high transcription of serine-threonine kinase B (*AKT1*) in leukocytes. These findings suggest Interleukin 6 (IL-6) and leptin take part in WAT-specific activation of *STAT3*. The binary logistic regression analysis confirmed an independent association of CVR with IL-6 in serum, and with *STAT3* in WAT. The study shows an association of CVR with transcriptional activity in WAT in female RA patients. It also emphasizes the importance of STAT3 regulatory circuits for WAT-related CVR in RA.

## 1. Introduction

Rheumatoid arthritis (RA) is strongly associated with increased frequency of cardiovascular (CV) disease, which remains the major cause of mortality in these patients [1,2]. Recent comprehensive epidemiological data revealed a 50% increase in CV death in RA patients compared with the general population [3]. Importantly, this excessive mortality in RA patients has neither declined in parallel with general population [4] nor decreased with advantages of modern anti-rheumatic treatment [5].

Several reasons have been outlined to explain this fact. On the one side, traditional CV risk (CVR) factors such as smoking and obesity are independently associated with development of RA, being a part of its pathogenesis [6,7]. On the other side, systemic inflammation with increased levels of C-reactive protein (CRP) and cytokines Tumor necrosis factor alpha (TNF-α), IL-6 and Interleukin 1 beta (IL-1β) is a hallmark of RA. Substantial role of inflammation in the development and progression of atherosclerosis has been connected to endothelial dysfunction that increases intima-media thickness and facilitates formation of atherosclerotic plaques [8]. Endothelial dysfunction has been found both in patients with long-standing RA [9] and in young RA patients with low disease activity and without traditional CVR factors [10]. Additionally, elevated levels of CRP are independently associated with systemic hypertension and insulin resistance increasing the risk of developing diabetes mellitus [11]. Consequently, contribution of these traditional CVR factors has only a limited value for estimation of new CV events in RA population.

Skewed gender distribution with predominance of women among RA patients is an important confounder for CVR assessment. Women with RA have more than 2-fold higher risk of developing myocardial infarction, even after adjusting for traditional CVR factors [12]. Similar to women in the general population, female RA patients have lower likelihood of presenting typical manifestations of CV diseases with angina symptoms and hence higher frequency of unrecognized myocardial infarction and sudden death [13]. This contributes to the fact that women are less likely to be referred for diagnostic and therapeutic procedures [14].

Adipose tissue attracts increasing attention as a risk factor for development of CV disease. It appears to be a remarkably complex organ controlling lipid metabolism and energy expenditure. Biological activity of adipose tissue is mediated by production and secretion of vast amounts of signaling molecules, which have profound effects on physiology of the whole organism. These molecules include classical cytokines such as TNF-α and IL-6, as well as adipocyte-specific soluble mediators leptin, resistin and adiponectin. Acting in an immediate neighborhood, these molecules define cellular composition of adipose tissue attracting macrophages and CD4^+^ T cells and controlling their differentiation. Circulating adipokines regulate appetite and reproductive function, insulin sensitivity in muscles and in liver. In the context of RA, pro-inflammatory and immune modulating properties of these signaling molecules are appreciated [15]. Indeed, higher serum levels of leptin, resistin and adiponectin are reported and commonly correlate with disease severity, inflammation markers and radiographic damage.

CV events, such as myocardial infarction and stroke, are often associated with high levels of leptin and resistin [16,17]. Pro-atherogenic properties of leptin and resistin are mediated through their ability to trigger expression of endothelial adhesion molecules and to enhance aggregation of platelets causing endothelial dysfunction. In addition to leptin-specific receptors, the pro-inflammatory and pro-atherogenic properties of leptin and resistin are carried out through activation of insulin-like growth factor 1 receptor (IGF1R) and Toll-like receptor (TLR) 4 signaling pathways [18,19,20]. Under physiological conditions, these two signaling pathways operate independently and mediate respectively metabolic and antibacterial functions. Under inflammatory conditions created by obesity and RA, activation of IGF1R and TLR4 synergize to trigger adaptive immune responses and T cell differentiation in the target adipose and synovial tissues [21,22,23,24].

The aim of the present study was to investigate the extent of transcriptional activity in white adipose tissue (WAT) and in leukocytes of the peripheral blood (WBC) and its impact on the assessment of CVR in female RA patients. The gene transcription analysis is focused on the inflammatory axis represented by Toll-like receptor 4 (*TLR4*), resistin (*RETN*) and transcription factor NF-κB p65 (*RELA*), and the metabolic axis represented by *IGF1R* and transcription factors *AKT1* and *STAT3*.

## 2. Results

### 2.1. Frequency of CVR Factors in the Study Cohort

The modified Systemic Coronary Risk Evaluation (mSCORE) in 182 women with RA identified the increased CVR in 93 of them (Table 1). As expected, the CVR group was older and had higher frequency of systemic hypertension and overweight compared to the group with no CVR. The CVR group was further characterized by accumulation of ever smokers (80%) and patients with diabetes mellitus. The RA-related CVR was represented by longer disease duration; 46% of the patients with CVR had the disease duration >10 years, while the percentage of patients with active disease (DAS28 > 3.2) and the frequency of RA-specific autoantibodies RF and ACPA was similar between the groups with CVR and with no CVR (Table 1). In total, 6 patients were treated with statins, all within the CVR group. Treatment with MTX, biologics and oral corticosteroids showed no differences between the two groups.

### 2.2. Expression of STAT3 is Enriched in WAT

**Figure 1:**
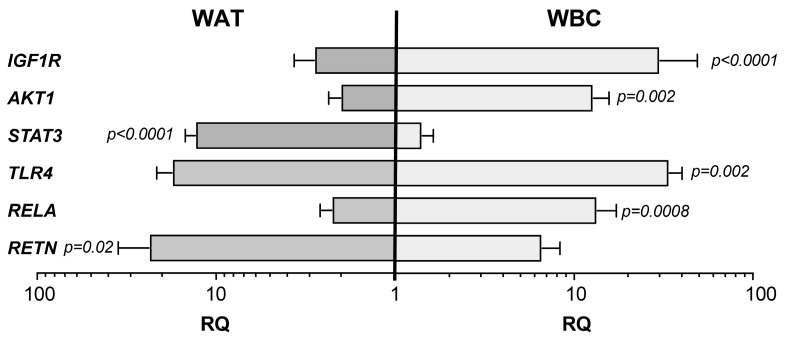
Gene expression analysis in leukocytes and in subcutaneous adipose tissue of patients with rheumatoid arthritis. Gene expression analysis was done in leukocytes of the peripheral blood (WBC, *n* = 95) and in subcutaneous adipose tissue (WAT, *n* = 83) of female RA patients by real-time PCR. Relative quantity (RQ) of expression for each gene was calculated in relation to the reference group that consisted of 11 young (mean age 38.4 years) non-smoking methotrexate-treated female RA patients with normal body weight and their disease in remission. Statistical comparison between gene expression in WBC and WAT samples was done with the nonparametric Mann-Whitney U test. The bars present mean with SEM. *IGF-1R*, insulin-like growth factor 1 receptor; *AKT1*, serine-threonine kinase 1; *STAT3*, signal transducer and activator of transcription 3; *TLR4*, Toll-like receptor 4; *RELA*, transcription factor p65; *RETN*, resistin.

The transcriptional activity of selected genes was measured in samples of WBC and WAT in RA patients (Figure 1). To enhance the value of CVR factors, the reference group for gene expression analysis was chosen among the young non-smoking methotrexate-treated female RA patients who had normal body weight and had the RA disease in remission.

### 2.3. Metabolic Axis IGF1R–AKT1 in CVR

Signaling through IGF1R activates the STAT3 and AKT1 pathways [25]. We asked if CVR is associated with alterations in IGF1R signaling in WBC and in WAT. The expression of *IGF1R* and *AKT1* showed comparable pattern with correlation in WAT (*r* = 0.62, *p* < 0.00001) and in WBC (*r* = 0.51, *p* < 0.00001). The transcription of both genes was higher in WBC as compared WAT (Figure 1).

**Figure 2:**
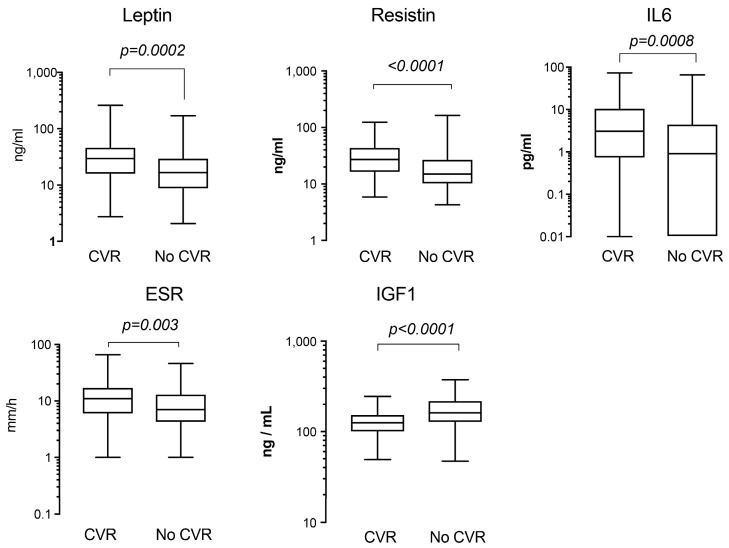
Serum levels of adipokines and inflammation markers in the rheumatoid arthritis (RA) patients with different cardiovascular risk. The cardiovascular risk (CVR) evaluation was done in 182 RA patients using flow charts corresponding to the low risk regions of Europe identified the groups with increased CVR (*n* = 93) and no CVR (*n* = 89). Statistical comparison between the groups was done with the Mann-Whitney U test. The boxes represent medians and Q1–Q3; whiskers show the min-max range. The presence of CVR was associated with high serum levels of leptin, resistin, IL-6 and erythrocyte sedimentation rate (ESR) and low serum levels of IGF1.

**Figure 3:**
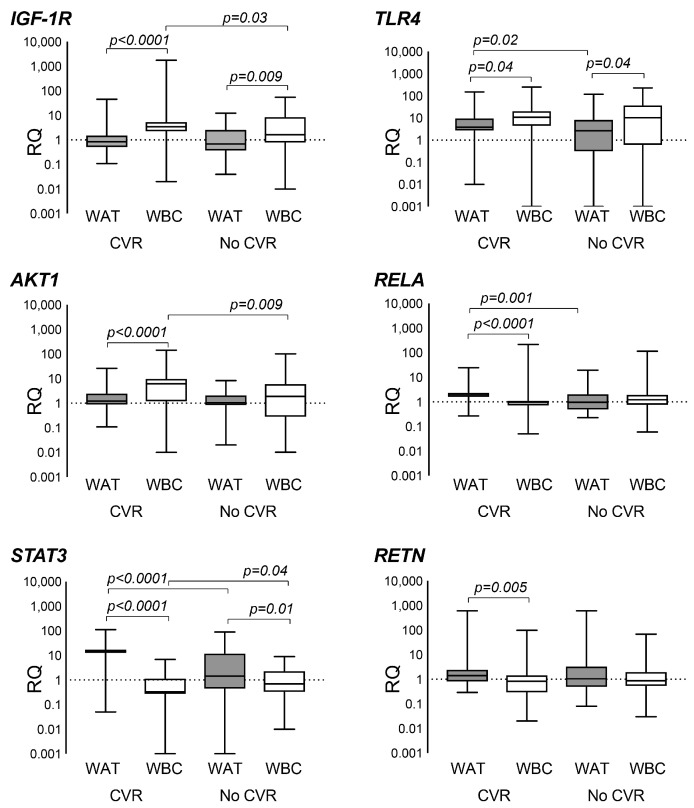
Gene expression analysis in leukocytes and in subcutaneous adipose tissue of patients with rheumatoid arthritis different in cardiovascular risk. Gene expression analysis was done in leukocytes of the peripheral blood (WBC, *n* = 95) and in subcutaneous adipose tissue (WAT, *n* = 83) of female RA patients by real-time PCR. Relative quantity (RQ) of expression for each gene was calculated in relation to the reference group that consisted of 11 young (mean age 38.4 years) non-smoking methotrexate-treated female RA patients with normal weight and their disease in remission. Statistical comparison between the group pairs was done with the nonparametric Mann-Whitney U test. The boxes represent medians, Q1–Q3; whiskers show min-max range. *IGF-1R*, insulin-like growth factor 1 receptor; *AKT1*, serine-threonine kinase 1; *STAT3*, signal transducer and activator of transcription 3; *TLR4*, Toll-like receptor 4; *RELA*, transcription factor p65; *RETN*, resistin.

The group with CVR had low serum levels of IGF1 and higher markers of systemic inflammation, as reflected by the increased serum levels of IL-6 and ESR, as well as the inflammation-associated WAT products, adipokines resistin and leptin (Figure 2). For *IGF1R*, this difference between WBC and WAT was significant both for the group with CVR and no CVR. For *AKT1*, the prevalent expression in WBC was clearly seen only in the CVR group (*p* = 0.013, Figure 3).

Transcription of *STAT3* is activated through cytokine receptors and through growth factor receptors and was significantly enriched in WAT compared to WBC (Figure 1). Higher levels of *STAT3* in WAT were also found in the patients with increased CVR (Figure 3).

*STAT3* transcription in WAT correlated significantly (*p* < 0.00001) to transcription factors NF-κB p65 (*RELA*; *r* = 0.69) and *AKT1* (*r* = 0.374), and to resistin (*RETN*, *r* = 0.486). *STAT3* in WAT correlated also to age (*r* = 0.567, *p* < 0.00001) and to the body fat content (*r* = 0.303, *p* = 0.015). We found no correlation of *STAT3* in WAT with inflammation, clinical activity of RA measured by DAS28 or the levels of pro-inflammatory cytokines IL-6 and IL-1β in serum.

*STAT3* transcription in WBC of the CVR group was lower compared to those with no CVR (Figure 3). *STAT3* in WBC correlated positively with pro-inflammatory *TLR4* (*r* = 0.460) and a negative correlation was found with NF-κB p65 (*RELA*, *r* = −0.445) and resistin (*RETN*, *r* = −0.58) (all, *p* < 0.00001).

IGF1 binding and subsequent activation of IGF1R leads to activation of AKT1. Serum levels of IGF1 were significantly lower in the CVR group (Figure 3). However, serum levels of IGF1 had no correlation to the transcription levels of *IGF1R*, neither in WBC nor in WAT. Adipokines are viewed as potential alternative ligands of IGF1R [26]. Surprisingly, we observed no clinically relevant correlations between serum levels of adipokines and *IGF1R* expression in WAT or WBC (all, *r* < 0.3).

### 2.4. Inflammatory Axis TLR4–NF-κB in CVR

TLR4-NF-κB axis is one of the most studied intracellular signaling pathways, which modulates inflammation by initiating production of TNF-α, IL-6 and IL-1β [27]. The pro-inflammatory effect of resistin is also mediated via TLR4 [19]. To investigate whether these intracellular pathways are connected to CVR, we compared the genes related to this pathway in WBC and WAT samples of female RA patients.

In concordance with the increased levels of pro-inflammatory IL-6, resistin and leptin, we found that the expression of *TLR4* was higher in WBC compared to WAT (Figure 2). However, CVR was associated with higher transcription of *TLR4* in WAT (Figure 3), while in WBC it was similar between the groups (not shown). Analogously, transcription of NF-κB p65 (*RELA*) was significantly higher in WBC, while the elevated *RELA* transcription was observed in WAT in the CVR group (Figure 3). *RELA* in WAT was correlated to *RETN* (*r* = 0.425), *TLR4* (*r* = 0.523), *STAT3* (*r* = 0.683), *AKT1* (*r* = 0.475) and *IGF1R* (*r* = 0.448) (all, *p* < 0.0001). *RELA* in WBC was correlated with serum resistin (*RETN*, *r* = 0.722) and with a decrease in *IGF1R* (*r* = −0.389), *AKT1* (*r* = −0.703) and *STAT3* (*r* = −0.428) (all, *p* < 0.00001).

### 2.5. Multivariate Regression Analysis of Parameters Associated with CVR in WAT

**Figure 4:**
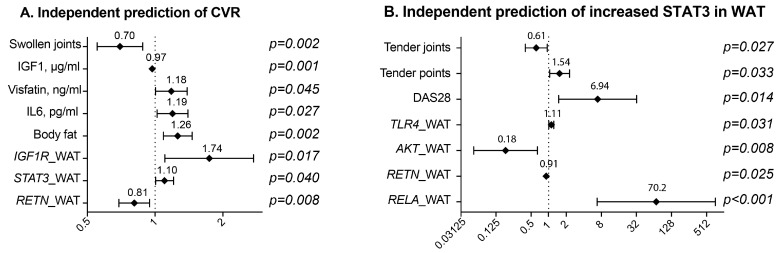
Parameters associated with cardiovascular risk (**A**) and with expression of *STAT3* in subcutaneous adipose tissue (**B**) of patients with rheumatoid arthritis. To identify the variables predicting increased CVR, a binary logistic regression with Wald backwards elimination was performed. The presence of CVR and was set as the dependent variable in the model A. The expression of *STAT3* in WAT within the upper 2/3 mRNA levels was considered increased and set as the dependent variable in the model B. Number of tender and number of swollen joints, number of tender fibromyalgia points (TP), erythrocyte sedimentation rate (ESR), disease activity score (DAS28), weekly dose of methotrexate (MTX), serum levels of IGF1, leptin, adiponectin, resistin, visfatin, IL-6, IL-1β, percentage of the body fat, and mRNA levels of *IGF1R*, *TLR4*, *AKT1*, *RETN*, *RELA* in white adipose tissue (WAT) were introduced as independent variables in the first step of the models. The importance of each variable was verified using Wald statistics and the variables of no importance (*p* > 0.1) were eliminated. The last step of the elimination analysis in the model A and model B is shown here and indicate the parameters independently associated with CVR and the increased expression of STAT3 in WAT.

To identify independent variables predicting the increased CVR in these female RA patients, a binary logistic regression with backwards elimination was performed. The mSCORE ≥ 1 indicated CVR and was set as the dependent variable in the model. Number of tender and number of swollen joints, number of tender fibromyalgia points, ESR, DAS28, weekly dose of MTX, serum levels of IGF1, leptin, adiponectin, resistin, visfatin, IL-6, IL-1β, percentage of the body fat, and mRNA levels of *IGF1R*, *STAT3*, *TLR4*, *AKT1*, *RETN*, *RELA* in WAT were introduced as independent variables in the first step of the model. The importance of each variable was verified using Wald statistics and the variables of no importance (*p* > 0.1) were eliminated. The last step of the elimination analysis is shown in Figure 4A. It revealed that the increased CVR was positively associated with the mRNA levels of *STAT3* in WAT, percentage of the body fat and serum levels of IL-6. The CVR was negatively associated with serum levels of IGF-1, mRNA of *RETN* in WAT and the number of swollen joints.

Since the binary regression analysis presented above identified *STAT3* in WAT as an independent predictor of CVR in RA females, we aimed to identify independent variables associated with its high transcription in WAT. The expression of *STAT3* in WAT within the upper 2/3 mRNA levels was considered high and set as dependent variable in the binary logistic regression model. The variables listed above were introduced as independent variables in the first step of the model and analyzed as previously. The last step of the elimination analysis is shown in Figure 4B. It indicated that the increased expression of *STAT3* in WAT was independently associated with clinical disease activity measured by DAS28 and the number of tender fibromyalgia points, whereas an inverse relation was found to the number of tender joints. The transcriptional activity in WAT presented by *RELA* (p65 NF-κB), *TLR4*, and low *AKT1* and *RETN* appeared to be associated with high *STAT3* in WAT (Figure 4B).

## 3. Discussion

In the present study, we analyzed the association of CVR with transcriptional activity in subcutaneous WAT and leukocytes of the peripheral blood of female patients with RA. The most noticeable finding in our study is the accumulation of *STAT3* in WAT of the patients with CVR compared to those with no CVR. The transcription of *STAT3* appeared to be tissue specific and found specifically in WAT being significantly less expressed in leukocytes of the peripheral blood.

STAT3 belongs to the family of transcription factors regulating tissue specific cellular differentiation and function by catalyzing Janus Kinase (JAK) signaling pathways. In RA, the activity of STAT3 in immune competent and joint-specific cells has been identified as an early and fateful event in the development of the disease [28]. Clinical relevance of STAT3 activation for RA is now confirmed by successful inhibition of JAK-STAT signaling reducing joint inflammation and skeletal damage [29]. In human and in rodent adipocytes, STAT3 controls adipogenesis acting upstream of peroxisome proliferator-activated receptor [30,31]. Inhibition of STAT3 in the adipose tissue is supposed to cause hypertrophy of adipocytes and increased mass of WAT, while activation of STAT3 has often a lipolytic effect [32]. Imbalance in lipid metabolism has been reported in the RA patients treated with JAK-STAT inhibitors being a cause of hypercholesterolemia and hypertriglyceridemia [33]. In concordance with these reports, our study showed a WAT-specific accumulation of *STAT3* in non-obese RA patients.

Several WAT products including IL-6 and leptin, may activate STAT3 by ligation of their receptors in para- and endocrine fashion. This is known to regulate insulin sensitivity, lipid metabolism and energy balance. Serum levels of IL-6 and leptin were significantly higher in patients with increased CVR and could be responsible for the observed accumulation of *STAT3* in WAT.

The results of our binary logistic regression analysis indicated serum levels of IL-6 and the expression of *STAT3* in WAT as independent CVR factors in RA female patients. High serum levels of IL-6 and leptin have previously been reported in connection to CVR in large epidemiological studies [34,35]. Similar to our cohort of RA patients, the increased levels of IL-6 are associated with traditional CVR factors as smoking, overweight and hypertension in apparently healthy women [36], and in elderly women with previous history of CV events [37]. Consistent with the lipolytic effect of IL-6, inhibition of IL-6R using tocilizumab in RA patients leads frequently to weight gain and dyslipidemia by increasing the levels of cholesterol and triglycerides and therefore potentially increasing CVR [38,39].

Ligation of IL-6R and trans-signaling of IL-6 through gp130 may also participate in the intracellular activation of NF-κB pathway, detectable in our study by the increased transcription of *RELA* and *TLR4* [40,41]. Alternatively, STAT3-independent activation of TLR4-NFκB pathway may be achieved by serum leptin [42]. The role of leptin in CVR varies and depends on the studied population [43,44]. It may have a protective effect in lean male patients with diabetes and be harmful in patients with metabolic syndrome and in women with SLE [45].

Several sources of potential bias should be considered. The study is done on the cohort of middle-aged RA females. On the one side, it reduces variability of the adipokine levels and of the transcriptional activity in WAT. On the other side, it represents the population of RA patients at risk for CV events due to high frequency of autoantibody positive, joint damaging and treatment resistant disease. However, hypercholesterolemia was identified to be the most prevalent CVR factor in this study present in 58% of the studied patients. The molecular mechanisms of hyperlipidemia in RA females are largely unknown. The impact of anti-rheumatic treatment on development of hypercholesterolemia is actively discussed [38,39,46]. Majority of the patients in our study are treated with MTX, which is known to decrease CVR [38]. The study revealed no significant increase in CVR for the patients on biologic treatment, which could potentially be due to a relatively low proportion (38%) of such patients in our study.

Taken together, the results of this study show an association of CVR with transcriptional activity in WAT in female patients with RA. It also emphasizes the importance of IL-6 and STAT3 regulatory circuit for WAT-related CVR in RA.

## 4. Materials and Methods

### 4.1. Patients

In total of 182 female patients with established RA diagnosis who fulfilled the American Rheumatism Association 1987 revised criteria [47] were included in the study between November 2011 and September 2013. Patients were recruited at the rheumatology units of the Sahlgrenska University Hospital in Gothenburg and the Northern Älvsborg Country Hospital in Uddevalla in Sweden. The patients were randomly chosen from the methotrexate (MTX)-treated patient cohorts. At the time of enrolment, all but 12 patients were treated with MTX. Sixty-eight patients had treatment with biologic drugs, (34 infliximab, 7 rituximab, 13 etanercept, 3 adalimumab, 5 golimumab, 5 tocilizumab, 1 abatacept). Twenty-five patients had MTX together with other disease modifying drugs (14 in combination with sulfasalazine, 7 in combination with hydrochloroquine and 4 in combination with both). Twenty-five patients were regular users of oral corticosteroids (median dose 5.0 mg/day). All patients completed structured questionnaire regarding their smoking habits, medication, and concomitant diseases. None of the patients had previous history of CV events. Six patients with type II diabetes were observed in the group with increased CVR and one patient with type I diabetes in low CVR group. At inclusion, all patients underwent clinical examination performed by experienced rheumatologists. The following clinical data were recorded: age, sex, body mass index (BMI), body fat content [48], disease duration. Disease activity score (DAS) was calculated based on assessment of 28 joints for tenderness and swelling and the erythrocyte sedimentation rate (ESR) (http://www.4s-dawn.com/DAS28/).

The study protocol was reviewed and approved by the Ethical Review Board of Gothenburg with permission code 659-2011. All methods used in this study were carried out in accordance with relevant Swedish guidelines and regulations and following the Good Clinical Practice. The informed written consent was obtained from all subjects prior to enrolment in the study.

### 4.2. Modified Systematic Coronary Risk Evaluation (mSCORE)

The assessment of cardiovascular risk (CVR) consisted of age, smoking (current or former smoker), and the presence of hypertension (blood pressure > 140/90 mm/Hg), diabetes mellitus, dyslipidemia (total cholesterol > 5 mmol/L) and overweight/obesity (BMI > 25 kg/m^2^). The SCORE index based on age, gender, smoking history, total serum cholesterol level and the systolic arterial blood pressure was constructed for each patient using a flow chart for the low risk regions of Europe [49,50]. The modified (m) SCORE index was obtained by multiplying the SCORE by 1.5 in the patients with disease duration above 10 years, and with RF or/and ACPA positivity [51].

### 4.3. Collection and Preparation of Blood and Adipose Tissue Samples

The blood samples were obtained after overnight fast. Blood specimens were drawn from the cubital vein directly into the vacuum tubes (Vacuette, Greiner Bio-One, Kremsmunster, Austria) containing serum clot activator, mixed thoroughly on BioMixer (Sarstedt, Numbrecht, Germany) coagulated for 3–4 h at room temperature and centrifuged at 2000× *g* for 10 min. The serum aliquots were carefully collected and stored at −80 °C until use.

The subcutaneous white adipose tissue (WAT) biopsies were obtained by needle aspiration of adipose tissue in the peri-umbilical area. In brief, a region 5 cm lateral from the umbilicus (either to the left or right side of the abdomen) was sterilized. A hypodermic needle 1.2 × 40 mm (18 G) was then adapted to a 20-mL syringe and the piston compressed. Approximately one-third of the length of the needle was inserted into the subcutaneous fat, and the needle piston was released maximally, thereby creating a vacuum. Tissue resistance was created by the physician gripping the abdominal wall with one hand while the other hand moving the needle throughout the tissue. Once the sample was aspirated into the syringe, the needle was withdrawn, and the piston was removed. Adipose tissue samples were preserved in AllProtect Tissue Reagent (Qiagen, Valencia, CA, USA) and stored in −80 °C.

### 4.4. Preparation of mRNA

PAXgene Blood RNA tubes (PreAnalytiX, Hombrechtikon, Switzerland) were used for blood sampling. Total RNA was extracted from the whole blood using PAXgene Blood RNA Kit (Qiagen) according to the manufacturer’s recommendations. RNA from fat tissue samples, stored in Allprotect Tissue Reagent (Qiagen), was prepared with RNeasy Plus Universal Kit (Qiagen). The concentration and quality of the RNA were evaluated with a NanoDrop spectrophotometer (Thermo Scientific, Waltham, MA, USA) and Experion (Bio-Rad laboratories Inc., Hercules, CA, USA). Four hundred ng RNA was used for cDNA synthesis using High Capacity cDNA Reverse Transcription Kit (Applied Biosystems, Foster City, CA, USA).

### 4.5. Gene Expression Analysis

Real-time polymerized chain reaction (RT-PCR) was performed on a ViiA™7 RT-PCR (Applied Biosystems, Foster City, CA, USA) using the customer designed multi-analyte array with primers for the *IGF1R*, *TLR4*, *AKT1*, *STAT3*, *RETN*, *RELA*, Actin B (*ACTB*) and RNA polymerase 2 (*POLR2A*) genes, and SYBR Green qPCR Mastermix (SA Biosciences, Frederick, MD, USA). The primer pairs can be found in the Table S1. Melting curves for each PCR were performed between 60 °C and 95 °C to ensure specificity of the amplified product. Expression levels of target genes were normalized to reference genes *ACTB* and *POLR2A*. Relative quantity (RQ) of expression for each gene was calculated in relation to the reference group using the ddCt method. The reference group consisted of 11 young (mean age 38.4 years) non-smoking normal body weight female RA patients, all treated with methotrexate (mean dose 16.4 mg/week) and had their disease in remission (mean DAS28 1.77).

### 4.6. Adipokine Measurement

The serum levels of resistin, adiponectin, leptin, visfatin, IGF1 and IL-6 were determined using specific sandwich ELISA kits (R&D Systems, Minneapolis, MN, USA). The minimum detectable concentrations were for resistin (DY1359, detection limit 10 pg/mL), adiponectin (DY1065, 62 pg/mL), leptin (DY398, 31 pg/mL), and for IL-6 (0.62 pg/mL). Visfatin was measured by an ELISA kit (AG-45A-0006TP-KI01, Adipogen Inc., Incheon, South Korea) with the minimal detection level of 125 pg/mL. All assays were performed according to the instructions from the manufacturers. ELISA plates were read with a dual wavelength on Spectramax 340 from Molecular Devices (Sunnyvale, CA, USA).

### 4.7. Other Serological Measures

The erythrocyte sedimentation rate (ESR) and serum levels of C-reactive protein (CRP), total cholesterol, IGF-1 were measured at the accredited Laboratory of Clinical Chemistry at the Sahlgrenska University Hospital according to clinical routines.

The measurements of RA specific antibodies to cyclic citrullinated peptides (ACPA) and rheumatoid factor (RF) were performed at the accredited laboratory of Clinical Immunology at the Sahlgrenska University Hospital. Detection of ACPA was done by automatic multiplex method for anti-CCP2 antibodies (BioRad, Hercules, CA, USA). The cut-off level above 3.0 AU/mL was set positive. RF was measured by rate nephelometric technology (Beckman Image 800, Beckman Coulter AB, Brea, CA, USA). The cut-off for RF positivity was set at 20 U/mL.

### 4.8. Statistical Analysis

Descriptive statistics for continuous variables are presented as the median with quartiles 1 and 3, and for categorical variables as the number and the percentage. Multiple imputation procedure was used to provide analysis of patterns of missing data. Less than 1% of data have been imputed. Univariate correlation between variables was examined by the Spearman’s correlation test. Any two factors with a correlation coefficient <0.3 were investigated for co-linearity. The material was dichotomized into the groups with CVR (mSCORE ≥ 1) and with no CVR (mSCORE < 1). For continuous variables, the difference was assessed by the Mann-Whitney U-test. The difference in frequency, sensitivity and specificity of calculations were performed using 2 × 2 table analysis and multiple chi-square tests (www.open-epi.org). Analyses were performed using Graph Pad Prism 6 for Microsoft Windows. Regression analyses were performed using IBM SPSS statistics 22. All tests were two tailed and *p* < 0.05 was considered statistically significant.

Predictive value of bioactive parameters for CVR and *STAT3* was calculated by composing binary logistic regression models where mSCORE and the upper 2/3 levels of *STAT3* in WAT were chosen as the dependent variables. The parameters not included in mSCORE, ESR, DAS28, BMI, swollen and tender joints, tender points, MTX dosage, body fat per cent, serum levels of leptin, resistin, visfatin, adiponectin, IGF1 and IL-6, mRNA levels of *TLR4*, *IGF1R*, *RETN*, *RELA*, *AKT1* in WAT were used as independent variables. Backward Wald elimination was used for predicting the dependence of parameters.

## Figures and Tables

**Table 1:** Clinical characteristics of RA female patients.

	No CVR *n* = 89	CVR *n* = 93	*p*-Value
Age, years	44 (35–59)	62 (58–64)	<0.0001
Total cholesterol, mmol/L, >5 mmol/L, *n* (%)	4.8 (4.1–5.5)	5.7 (5.2–6.5)	<0.0001
	31 (35)	74 (80)	<0.0001
Systolic blood pressure, >140 mmHg, *n* (%)	2 (2)	26 (28)	<0.0001
Diabetes mellitus	1	6	0.1
Disease duration, years, >10 years, *n* (%)	6 (3–10)	10 (6–18)	0.0002
	20 (22)	42 (45)	0.0013
RF and/or ACPA positive, *n* (%)	78/89 (88)	86/95 (92)	0.54
DAS28 > 3.2, *n* (%)	38/84 (45)	42/88 (48)	1
Body fat content, %	33 (30–37)	40 (36–43)	0.005
BMI, kg/m^2^	23.6 (21–26)	26.3 (23–29)	0.0005
BMI > 25 kg/m^2^, *n* (%)	33/88 (37)	58/93 (62)	0.0007
ESR, mm/h	7 (4.2–13.0)	11 (6.0–17.0)	0.002
Methotrexate, mg/week	17.5 (13–20)	17.5 (15–20)	0.74
Current/former smoker, *n* (%)	41/89 (46)	74/93 (80)	<0.0001

The cardiovascular risk (CVR) evaluation by flow charts corresponding to the low risk regions of Europe identified the patients with cardiovascular risk (CVR, mSCORE ≥ 1, *n* = 93) and no CVR (mSCORE < 1, *n* = 89). The continuous variables are presented as median (Q1–Q3). Statistical comparison between the groups was done with the Mann-Whitney U test, and the chi-square analysis. ESR, erythrocyte sedimentation rate; RF, rheumatoid factor, ACPA, antibodies to citrullinated peptides; DAS28, disease activity score based on assessment of 28 joints; BMI, body mass index.

## Supplementary Materials

Supplementary materials can be found at www.mdpi.com/1422-0067/18/11/2410/s1.

## Abbreviations

RA	rheumatoid arthritis
mSCORE	modified Systemic Coronary Risk Evaluation
IQR	interquartile range
RF	rheumatoid factor
ACPA	antibodies against citrullinated peptides
DAS28	28 joint count Disease Activity Score
BMI	body mass index
CVR	cardiovascular risk
STAT	signal transducer and activator of transcription
RELA	transcription factor p65
NF-κB	nuclear factor kappa B
IGF1	insulin-like growth factor 1
IGF1R	insulin-like growth factor 1 receptor
AKT1	serine-threonine kinase 1
WAT	white adipose tissue
WBC	white blood cells
TLR	Toll-like receptor
OR	Odds ratio
CI	Confidence interval
