# Supplementary material for: High Expression of STAT3 in Subcutaneous Adipose Tissue Associates with Cardiovascular Risk in Women with Rheumatoid Arthritis
Source: Int J Mol Sci. 2017 Nov 13;18(11):2410. doi: 10.3390/ijms18112410 (PMC5713378; doi:10.3390/ijms18112410)
Supplement: Supplementary file 1 [file ijms-18-02410-s001.pdf]

Supplementary Table S1

Primers used for qPCR analysis of WBC and WAT specimens.

Assay on pre primed plate from SA Biosciences

	Cat.nr. (SABioscience, Qiagen)
IGF1R	PPH00350F
STAT3	PPH00708F
TLR4	PPH01795F
AKT1	PPH00088B
RETN	PPH02309B
RELA	PPH01812B
ACTB	PPH00073G
POLR2A	PPH13508E
